# Occurrence, genetic diversity and zoonotic potential of *Blastocystis* sp. in forest musk deer (*Moschus berezovskii*) in Southwest China[Fn FN1]

**DOI:** 10.1051/parasite/2022037

**Published:** 2022-07-14

**Authors:** Shanyu Chen, Wanyu Meng, Xianpeng Shi, Yijun Chai, Ziyao Zhou, Haifeng Liu, Zhijun Zhong, Hualin Fu, Suizhong Cao, Xiaoping Ma, Liuhong Shen, Lei Deng, Guangneng Peng

**Affiliations:** 1 The Key Laboratory of Animal Disease and Human Health of Sichuan Province, College of Veterinary Medicine, Sichuan Agricultural University Chengdu 611130 Sichuan People’s Republic of China; 2 Laboratory of Molecular and Cellular Parasitology, Healthy Longevity Translational Research Programme and Department of Microbiology and Immunology, Yong Loo Lin School of Medicine, National University of Singapore Singapore 117545 Singapore

**Keywords:** *Blastocystis* sp., Zoonotic potential, Forest musk deer, Prevalence, China

## Abstract

*Blastocystis* sp. is a common anaerobic protist with controversial pathogenicity that can infect various animals and humans. However, there are no reports of *Blastocystis* sp. infections in forest musk deer (*Moschus berezovskii*). The present study was designed to examine the occurrence, subtype distribution and genetic characterization of *Blastocystis* sp. in forest musk deer in southwestern China, and to assess the potential for zoonotic transmission. A total of 504 fresh stool samples were collected from captive forest musk deer in four distinct areas of southwestern China. Overall, 14.7% of the forest musk deer (74/504) were found to be infected with *Blastocystis* sp. The highest occurrence of *Blastocystis* sp. was observed in Dujiangyan (27.5%), followed by Maerkang (23.3%). The occurrence of *Blastocystis* sp. was 7.9% and 4.1% in Shimian and Hanyuan, respectively. Significant differences in the occurrence of *Blastocystis* sp. among different areas were observed (*p* < 0.05), while we did not observe significant differences among animals of different age and sex (*p* > 0.05). Two known zoonotic subtypes (ST1 and ST5) and three animal-predominant subtypes (ST10, ST13, and ST14) were identified, of which ST10 was the most common (36/74, 48.6%). Our findings highlight that forest musk deer may be potential reservoirs of zoonotic human *Blastocystis* sp. infections.

## Abstract (fr)

*Blastocystis* sp. est un protiste anaérobie commun, de pathogénicité controversée, et qui peut infecter divers animaux et les humains. Cependant, aucun cas d’infection par *Blastocystis* sp. n’a été rapporté chez le cerf porte-musc (*Moschus berezovskii*). La présente étude a été conçue pour examiner la présence, la distribution des sous-types et la caractérisation génétique de *Blastocystis* sp. chez le cerf porte-musc du sud-ouest de la Chine et pour évaluer son potentiel de transmission zoonotique. Au total, 504 échantillons de selles fraîches ont été prélevés sur des cerfs porte-musc captifs dans quatre régions distinctes du sud-ouest de la Chine. Dans l’ensemble, 14,7 % (74/504) des cerfs porte-musc se sont avérés infectés par *Blastocystis* sp. La plus forte occurrence de *Blastocystis* sp. a été observée à Dujiangyan (27,5 %), suivi de Maerkang (23,3 %). La présence de *Blastocystis* sp. était respectivement de 7,9 % et 4,1 % à Shimian et Hanyuan. Des différences significatives dans la présence de *Blastocystis* sp. entre les différentes zones ont été observées (*p* < 0,05), alors que nous n’avons pas observé de différences significatives entre les animaux d’âge et de sexe différents (*p* > 0,05). Deux sous-types zoonotiques connus (ST1 et ST5) et trois sous-types à prédominance animale (ST10, ST13 et ST14) ont été identifiés, dont ST10 était le sous-type le plus courant (36/74, 48,6 %). Nos découvertes mettent en évidence que le cerf porte-musc forestier peut être un réservoir potentiel d’infections à *Blastocystis* sp.

## Introduction

*Blastocystis* sp. belongs to the phylum Stramenopiles and is a common unicellular intestinal parasite of various animals. Generally, *Blastocystis* sp*.* is transmitted via the fecal-oral route, which is the primary mode of transmission [22, 23, 44]. Several studies have shown that humans are susceptible to zoonotic *Blastocystis* sp. [31, 50]*.* Epidemiological surveys estimate that the parasite has colonized between one and two billion people worldwide [34]. However, the pathogenicity of *Blastocystis* sp. is still uncertain, although some studies have demonstrated possible associations of the parasite to a variety of gastrointestinal disorders, such as irritable bowel syndrome (IBS) and inflammatory bowel disease (IBD) [7, 13, 19]. In contrast, some studies demonstrated that *Blastocystis* sp. is a common commensal microorganism in the human gut, associated with increased diversity of gut microbiota [4, 6, 46].

Based on analysis of the small subunit (SSU) rRNA gene of *Blastocystis* sp., at least 28 subtypes (ST1–ST17, ST21, ST23–ST32) have been confirmed in humans and in a variety of animals worldwide [17, 27, 28, 42]. Among them, ST1 to ST9 and ST12 are known to infect humans, while ST1 to ST4 account for more than 90% of human *Blastocystis* sp. infections [25, 43]. Interestingly, the prevalence of different subtypes seems to vary greatly among different regions and countries [10], and different subtypes demonstrate remarkably diverse biological characteristics, such as pathogenicity, drug resistance, and effects on microbiota [1, 32, 53].

The forest musk deer (*Moschus berezovskii*) is a small ruminant unique to Asia and belongs to the Moschidae family [12]. Musk deer (*Moschus* spp.) are an endangered species currently considered class I-protected animals in China. The forest musk deer is the largest musk deer species in China, mainly distributed in Guizhou and Sichuan province [16, 51]. It has been determined that forest musk deer can harbor several zoonotic intestinal pathogens (e.g., *Enterocytozoon bieneusi* and *Giardia duodenalis*) and have the ability to transmit these organisms to humans [39]. However, there are no studies focusing on the isolation of *Blastocystis* sp. from forest musk deer, and whether it is an infection reservoir for other animals and humans remains unclear. In this study, we explored the prevalence and subtype distribution characteristics of *Blastocystis* sp. in forest musk deer for the first time, emphasizing the potential threat of zoonotic transmission.

## Materials and methods

### Ethics statement

This study was performed in accordance with the recommendations of the Guide for the Care and Use of Laboratory Animals of the Ministry of Health, China. As only fecal samples collected after spontaneous defecation of forest musk deer were analyzed, this study did not require full Animal Ethics Committee approval in accordance with Chinese law. No animals were harmed during the sampling process. Permission was obtained from farm owners and managers before collection of fecal specimens.

### Sample collection

**Figure 1:**
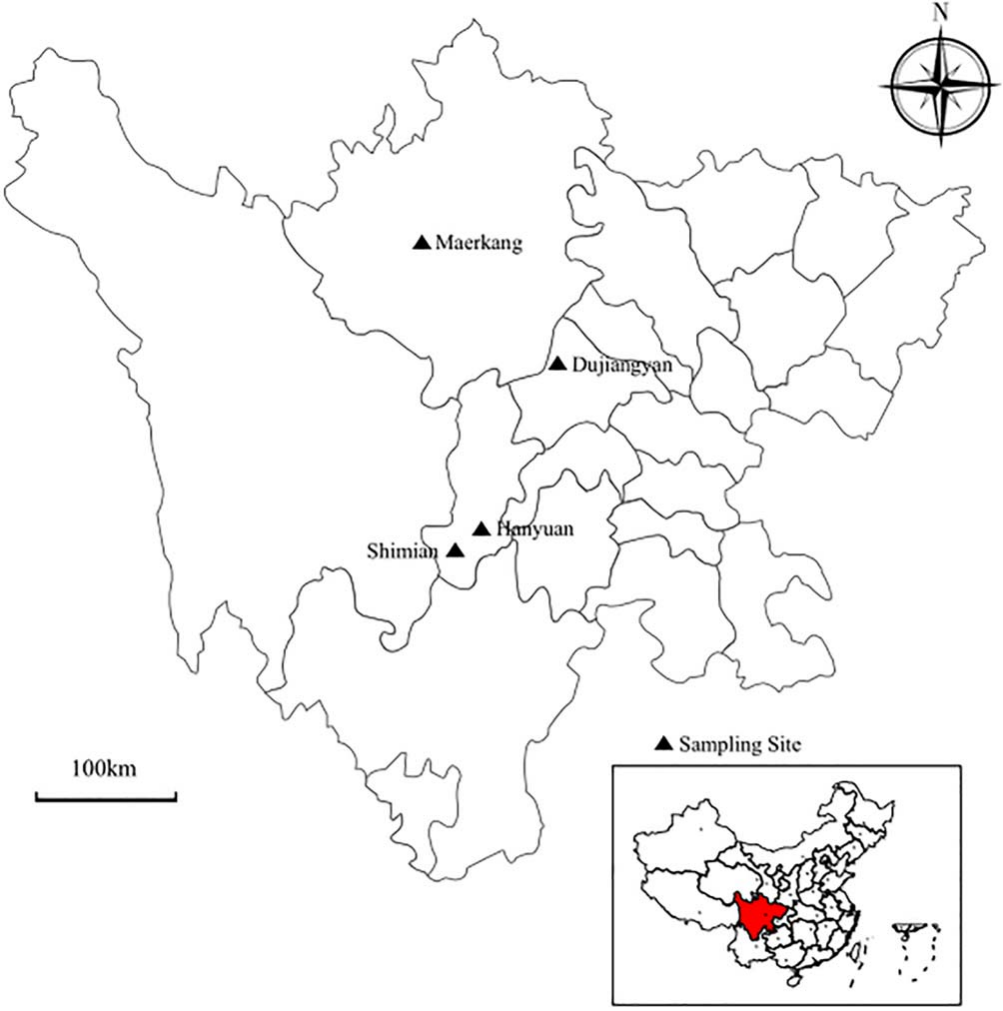
Locations of the sampled sites (filled triangle) in Sichuan Province, Southwestern China.

A total of 504 fecal samples from captive forest musk deer was collected from four areas of Sichuan province between August and September 2020 (Fig. 1), with 139 samples collected in Shimian (29°16′ N, 102°20′ E) at an altitude of 2572 m, 144 in Hanyuan (29°29′ N, 102°37′ E) at an altitude of 1076 m, 131 in Dujiangyan (31°01′ N, 103°35′ E) at an altitude of 739 m, and 90 in Maerkang (31°53 N, 102°07′ E) at an altitude of 2526 m (Table 1). The forest musk deer breeding farms were cleaned the night before sampling, and each individual was kept in a separate enclosure so that the fresh feces of each individual could be collected the following morning. All fecal samples were collected by laboratory staff or farmers trained in sample collection, and strict controls were implemented to minimize potential contamination between samples. Approximately 5–10 g of fresh fecal samples were collected using sterile disposal latex gloves after defecation of the forest musk deer, stored in individual plastic bags, with gender, age, and number recorded. During the sample collection process, only the middle layer of feces was collected to avoid contamination. All samples were immediately stored in liquid nitrogen for transportation back to the laboratory and later stored at −80 °C until processing. The animals that had been sampled exhibited no obvious clinical signs.

**Table 1:** Factors associated with the prevalence of *Blastocystis* in forest musk deer in China.

Factors	No. of positive/overall	Prevalence (95% CI)	OR (95% CI)	*P* value
Locations				
Shimian	11/139	7.9 (3.4–12.4)	Reference	Reference
Hanyuan	6/144	4.1 (0.9–7.4)	0.5 (0.2–1.4)	0.192
Dujiangyan	36/131	27.5 (19.8–35.1)	4.4 (2.1–9.1)	0
Maerkang	21/90	23.3 (14.6–32.1)	3.5 (1.6–7.8)	0.002
Sex				
Female	29/243	11.9 (7.9–16.0)	Reference	Reference
Male	45/261	17.2 (12.7–21.8)	1.5 (0.9–2.5)	0.094
Age (years)				
≦ 1.5	19/119	16.0 (9.3–22.5)	Reference	Reference
> 1.5	55/385	14.3 (10.8–17.8)	0.9 (0.5–1.5)	0.651
Total	74/504	14.7 (11.6–17.8)		

### DNA extraction

Fecal samples were sieved and washed with distilled water three times by centrifugation at 3000× *g* for 10 min. Genomic DNA was extracted using a QIAamp Fast DNA Stool Mini Kit (Qiagen, Germany), according to the manufacturer’s instructions. Both negative and positive control stools were included. The DNA was eluted in 200 μL of buffer and stored at –20 °C until use, and the quality of the DNA was verified using NanoDrop (Thermo Fisher Scientific, Carlsbad, CA, USA).

### PCR amplification

PCR amplification of the barcode region (a fragment of ~600 bp) of the SSU rRNA gene was used to screen all DNA preparations to identify *Blastocystis* sp. The cycling parameters and primers were the same as previously described by Scicluna et al. [35]. Taq PCR Master Mix (Sangon Biotech Co., Ltd., Shanghai, China) was used for all PCRs. All PCR tests included positive and negative controls and were performed in triplicate. The PCR products were subjected to 1.5% agarose gel electrophoresis and visualized by staining with SYBR Safe DNA Gel Stain (Thermo Fisher Scientific).

### Nucleotide sequencing and analysis

A QIAQuick Gel Extraction Kit (Qiagen) was used to purify PCR products from agarose gel, according to the manufacturer’s instructions. The expected product size was ~600 bp. All positive PCR products were bidirectionally sequenced at the BioSune Biotechnology Company (Shanghai, China); A BigDye Terminator v3.1 Cycle Sequencing kit (Applied Biosystems, Waltham, MA, USA) was used. The consensus sequences obtained in our study were submitted to BLAST searches (http://www.ncbi.nlm.nih.gov/blast/), then aligned and analyzed. Reference sequences were downloaded from the GenBank database (http://www.ncbi.nlm.nih.gov); we then used Clustal X 2.0 (http://www.clustal.org/) to identify the subtypes of *Blastocystis* sp. The nucleotide sequences generated in this study were deposited in GenBank with the accession numbers OK445532–OK445537, and OK445663–OK445665.

### Partial phylogenetic analysis

To evaluate the genetic relationship among the sequences of *Blastocystis* sp. genotypes obtained in this study and those identified previously, MEGA 6 software (http://www.megasoftware.net/) was used to construct a neighbor-joining tree for partial phylogenetic analysis. The Kimura 2-parameter model was used to calculate the evolutionary distances. Undefined positions were removed from the alignment before partial phylogenetic analysis, and the alignment was trimmed by MEGA 6. Finally, we assessed the reliability of the trees by Bootstrap analysis (with 1000 replicates).

### Statistical analysis

Variations in the prevalence of *Blastocystis* sp. (y1) in forest musk deer according to geographical location (x1), sex (x2), and age (x3) were analyzed by binary logit model using SPSS 22 (https://www.ibm.com/analytics/spss-statistics-software). *P*-value < 0.05 represented statistical significance (Table 1).

## Results

### Prevalence of *Blastocystis* sp. in forest musk deer

A total of 504 fecal samples (144 Hanyuan, 139 Shimian, 131 Dujiangyan, and 90 Maerkang) were screened by PCR amplification to identify *Blastocystis* sp. The overall prevalence of *Blastocystis* sp. in forest musk deer was 14.7% (74/504). The highest prevalence was in Dujiangyan at 27.5% (36/131), followed by Maerkang 23.3% (21/90), Shimian 7.9% (11/139), and Hanyuan 4.1% (6/144) (Table 1). There were significant differences in prevalence between the four areas (*p* < 0.05). The prevalence of *Blastocystis* sp. among females and males were 48.2% and 51.9%, respectively but there was no significant difference between them (*p*s > 0.05). Similarly, the differences in prevalence of *Blastocystis* sp. among forest musk deer of different ages were not statistically significant (*p* > 0.05)*.*

### Subtype distributions of *Blastocystis* sp. in forest musk deer

Five subtypes of *Blastocystis* sp. were identified from 74 positive samples, including two potentially zoonotic STs (ST1, ST5) and three animal-specific STs (ST10, ST13, ST14). Although Sanger sequencing cannot identify the subtypes involved in mixed infection and only identified the dominant subtypes in the samples, we believe that there were no mixed infections because there were no ambiguous peaks in the electropherograms. ST10 (36/74) was the dominant subtype found in the forest musk deer examined, followed by ST5 (18/74), ST13 (10/74), ST14 (6/74) and ST1 (4/74) (Table 2). Interestingly, ST1 was found only in forest musk deer in Shimian.

**Table 2:** Subtype distributions from different locations.

*Blastocystis* sp. STs (accession number)	Shimian	Hanyuan	Dujiangyan	Maerkang
ST1 (OK445532)	2			
ST1 (OK445533)	2			
ST5 (OK445534)			13	5
ST10 (OK445535)	2	3	17	8
ST10 (OK445536)				2
ST10 (OK445537)			2	2
ST13 (OK445663)	2	1		
ST13 (OK445664)	3	2		2
ST14 (OK445665)			4	2
Total	11	6	36	21

### Genetic characteristics of *Blastocystis* sp. subtypes

Analysis of the SSU rRNA gene revealed that four sequences of ST1 isolate contained two representative sequences, the sequences OK445532 (*n* = 2) and OK445533 (*n* = 2). They have 99.82% and 99.65% similarity to the ST1 sequence isolated from humans (MK782501), with one and two nucleotide substitutions, respectively. ST5 sequences (*n* = 18) showed 100% identity to that of alpaca in China (MN382283). ST10 isolates contained three representative sequences, the sequences OK445536 (*n* = 2) and OK445537 (*n* = 4) were identical to sheep from Iran (MW426240) and cattle from Malaysia (MG831508), respectively. The remaining sequence OK445535 (*n* = 30) showed 99.82% similarity to sika deer from China (MK930355) and white-tailed deer from the USA (MZ267679) with one nucleotide substitution. ST13 isolates (*n* = 10) contained two representative sequences, the sequence OK445663 (*n* = 3) showed 100% identity to a sequence that was isolated from crested deer in China (MT889741), and the sequence OK445664 (*n* = 7) showed 99.82% similarity to a sambar sequence from South Korea (MT114848) with one nucleotide substitution. ST14 sequences (*n* = 6) exhibited 100% identity to that of sheep in China (MT672788) and the Czech Republic (MT039559).

### Partial phylogenetic analysis of *Blastocystis* sp.

**Figure 2:**
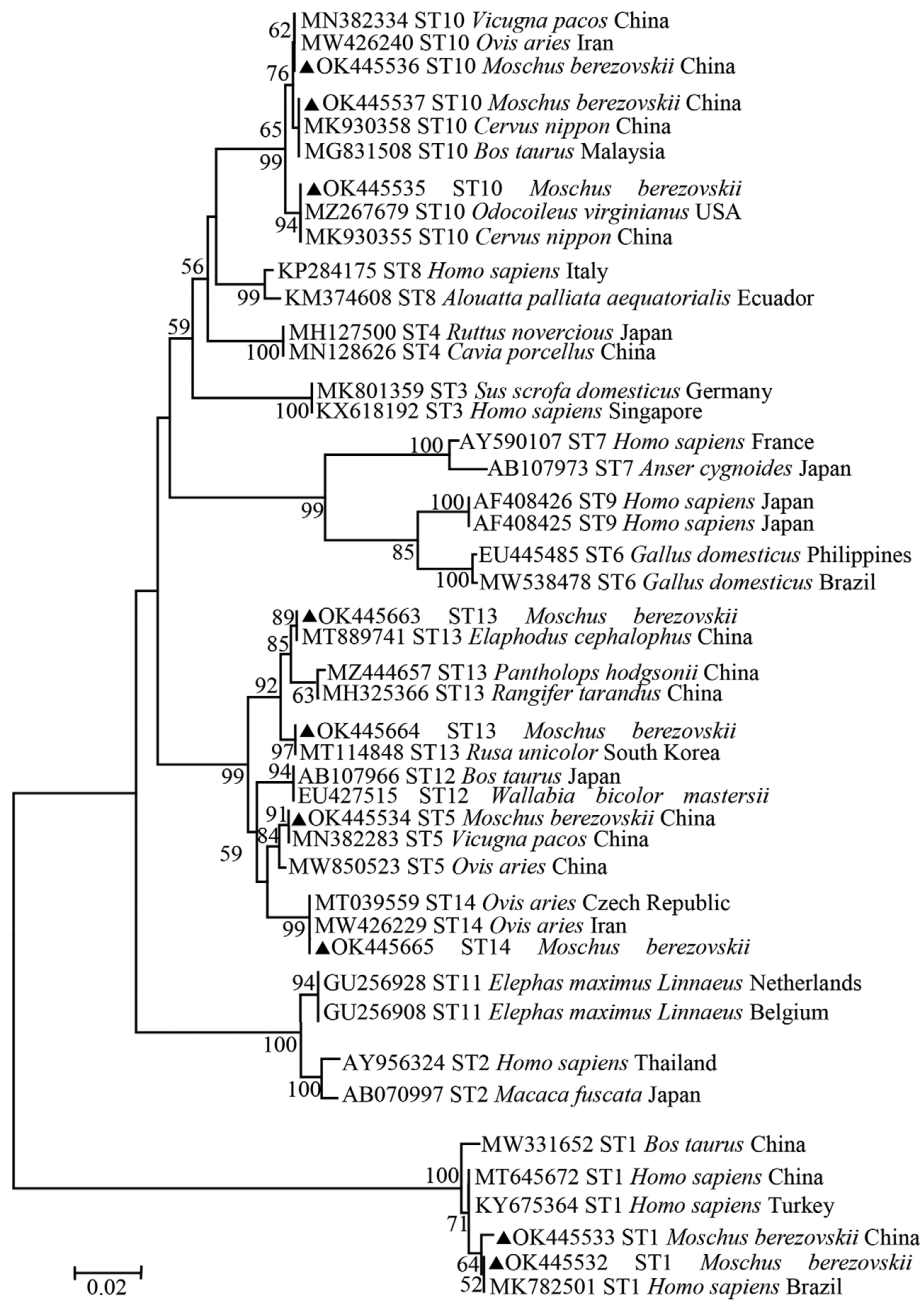
Partial Phylogenetic relationships among nucleotide sequences of *Blastocystis* sp. partial small subunit ribosomal RNA (SSU rRNA) genes. The neighbor-joining method was used to construct the trees by the Kimura-2-parameter model. The numbers on the branches are percent bootstrap support values from 1000 replicates; only values of more than 50% are shown on the tree. Each sequence is identified by its accession number, subtype, host origin, and country. Sequences marked with black triangles are representative sequences identified in this study.

Nine representative sequences were obtained from the 74 *Blastocystis* sp. isolates in this study. These newly identified sequences showed high similarity to reference sequences of *Blastocystis* sp. in GenBank, and belong to ST1, ST5, ST10, ST13 and ST14. The ST1 found in this study clustered together with sequences originating from humans and cattle. ST5 is grouped with sequences that are mainly from sheep and alpaca. ST10 clustered with sequences from sheep, sika deer, alpaca, cattle, and white-tailed deer, while ST13 grouped with sequences isolated from crested deer, Tibetan antelope, reindeer and sambar. ST14 formed a clade with sequences from sheep (Fig. 2).

## Discussion

*Blastocystis* sp. is one of the most common parasites and is distributed globally. Epidemiological studies of *Blastocystis* sp. in wild ruminants, such as takin, bushbuck, red deer, fallow deer, white-lipped deer, giraffe and reindeer, have been reported, but there is no research on captive forest musk deer [18]. The prevalence of *Blastocystis* sp. in forest musk deer examined in this study was 14.7% (74/504), which is lower than that previously found in wild takin in China (57.1%, 28/49) [56], wild Père David’s deer in China (56.3%, 72/128) [29], wild Korean water deer in Korea (40.8%, 51/125) [21], farmed Alpine musk deer in northwestern China (39.8%, 80/201) [49], farmed goats in Malaysia (30.9%, 73/236) [45], and farmed camels in Libya (24%, 47/196) [3]. However, the prevalence is higher than that observed in farmed sika deer in northeastern China (14.6%, 12/82) [47], wild reindeer in China (6.73%, 7/104) [48], farmed sika deer in northern China (0.8%, 6/760) [30], and farmed goats in Nepal (0.75%, 3/400) [15]. The reason for the different prevalence of *Blastocystis* sp. may be due to the captive conditions, management methods, the size of the examined samples, the animal species and different countries.

In this study, we found no statistical differences in the prevalence of *Blastocystis* sp. among females and males, nor between age groups (*p* > 0.05). However, the infection prevalence in forest musk deer examined was significantly different depending on the geographical area of origin in Sichuan Province (*p* < 0.05), with the highest prevalence (27.5%) in Dujiangyan and the lowest (4.1%) in Hanyuan. Previous reports have observed similar differences in the prevalence of this protist in cattle between different regions of China [57]. The different *Blastocystis* sp. prevalence may be related to the farm management methods and sanitary conditions in distinct regions. The higher prevalence in Dujiangyan and Maerkang is due to the lack of good immunization programs and deworming, as well as relatively poor hygiene conditions.

Five *Blastocystis* sp. subtypes (ST1, ST5, ST10, ST13 and ST14) were identified in 74 *Blastocystis* sp.-positive fecal samples from forest musk deer (Table 2). Maloney et al. used PCR and next generation amplicon sequencing to determine the occurrence and subtypes of *Blastocystis* sp. in white-tailed deer (*Odocoileus virginianus*) [27]. Ten previously reported subtypes (ST1, ST3, ST4, ST10, ST14, ST21, and ST23–ST26) and two novel subtypes (ST30 and ST31) were identified [27]. However, except for ST1, ST10 and ST14, none of the other subtypes found in white-tailed deer (*Odocoileus virginianus*) were identified in this study. Similarly, Ni et al used amplification of the SSU rDNA gene to confirm the presence of *Blastocystis* sp. infection in Père David’s deer (*Elaphurus davidianus*) in the National Nature Reserve of Shishou, Hubei Province of China [29]. Five known subtypes, which consisted of one zoonotic subtype (ST10) and four ruminant-specific subtypes (ST21, ST23, ST25, and ST26), were identified [29]. However, except for ST10, the other subtypes found in Père David’s deer were not found in forest musk deer in this study.

In China, ST10 is the main subtype in animal infection [26, 33, 38, 56, 57], followed by ST5 [37], and the results of the present study are in line with this conclusion. Zoonotic STs can be transmitted between humans and animals, and some animal-origin STs are linked to human infections [24, 36, 58]. One potentially zoonotic subtype identified in this study, ST1, has been reported as one of the most widespread subtypes in humans [50]. Previous studies have shown that the *Blastocystis* sp. subtypes ST1, ST2 and ST3 were commonly identified in primate hosts [11, 31, 55]. ST1 is also found in ruminants around the world, such as sika deer in China [9] and white-tailed deer in the United States [27]. Subtype ST1 was found in forest musk deer in this study (Table 2). Interestingly, the ST1 subtype variant detected in this study from Shimian farm forest musk deer showed high similarity with known sequences from humans in China (MT645672), emphasizing that these STs have the potential for zoonotic transmission. ST5, another zoonotic subtype detected in this study, is the dominant subtype infecting hoofed animals like pigs and cattle worldwide [2, 5, 37, 50, 54]. Additionally, ST5 has also been detected in humans with animal contact history, demonstrating that this subtype has zoonotic transmission risk [41, 52]. For instance, ST5 has been detected in both pigs and humans in Jiangxi province, where children and pigs sometimes share common outdoor areas [52].

In contrast, although ST10 has rarely been detected in humans, it is very prevalent in Artiodactyla [3, 18, 50], such as waterbuck in Bangladesh [24], takin, yak, bushbuck, eland and reindeer in China [48, 56], and roe deer in Denmark [40]. The results of this study indicate that ST10 was the most prevalent *Blastocystis* sp. ST in forest musk deer, which is consistent with the previously reported dominance of ST10 in Artiodactyla [56]. Moreover, as ST10 was identified in all four forest musk deer farms, ST10 distribution is not limited to certain geographic locations and has a wider range than reported [3]. Surprisingly, ST13, relatively rare in this study, was also detected in a mouse deer in the United Kingdom [3] and Java mouse-deer in France [8]. So far, ST14 has mostly been detected in Artiodactyla such as sheep, camels, mouflon and cattle [3, 14, 26, 56]. A recent study reported that ST10 and ST14 can also infect humans [20].

## Conclusions

This study determined the occurrence, subtype distribution and genetic characteristics of *Blastocystis* sp. for the first time in captive forest musk deer in China. The data showed that five subtypes of *Blastocystis* sp. including two zoonotic subtypes (ST1 and ST5) can infect forest musk deer. These findings provide fundamental data for monitoring and exploring the transmission routes of *Blastocystis* sp. between forest musk deer and humans.

**Abbreviations**
PCR: Polymerase chain reaction;
STs: Subtypes;
SSU rRNA: Small subunit ribosomal RNA;
IBS: Irritable bowel syndrome;
IBD: Inflammatory bowel disease;
ORs: Odds ratios.

## Data Availability

The nucleotide sequences generated in the present study have been deposited in GenBank (https://www.ncbi.nlm.nih.gov/) under accession numbers OK445532–OK445537, and OK445663–OK445665. The datasets used and/or analyzed during the current study are available from the corresponding author on reasonable request.
